# Packaging signal of influenza A virus

**DOI:** 10.1186/s12985-021-01504-4

**Published:** 2021-02-17

**Authors:** Xiuli Li, Min Gu, Qinmei Zheng, Ruyi Gao, Xiufan Liu

**Affiliations:** Animal Infectious Disease Laboratory, College of Veterinary Medicine, Yangzhou University, 48 East Wenhui Road, Yangzhou, Jiangsu China

**Keywords:** Influenza A virus, Packaging signals, Incorporation, Reassortment, RNA-RNA interaction

## Abstract

Influenza A virus (IAV) contains a genome with eight single-stranded, negative-sense RNA segments that encode 17 proteins. During its assembly, all eight separate viral RNA (vRNA) segments are incorporated into virions in a selective manner. Evidence suggested that the highly selective genome packaging mechanism relies on RNA-RNA or protein-RNA interactions. The specific structures of each vRNA that contribute to mediating the packaging of the vRNA into virions have been described and identified as packaging signals. Abundant research indicated that sequences required for genome incorporation are not series and are varied among virus genotypes. The packaging signals play important roles in determining the virus replication, genome incorporation and genetic reassortment of influenza A virus. In this review, we discuss recent studies on influenza A virus packaging signals to provide an overview of their characteristics and functions.

## Background

**Fig. 1:**
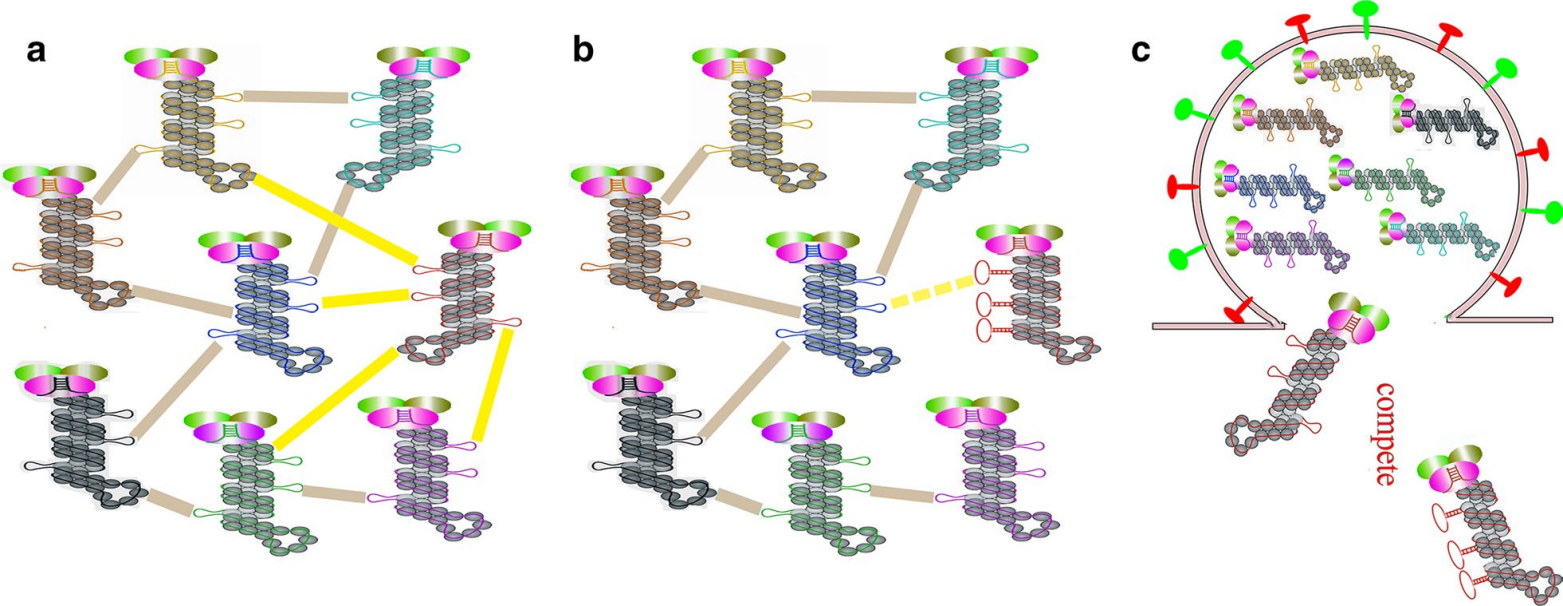
A model for selective incorporation of IAV genome. Different vRNAs are shown as lines of varied colors (red, green, yellow, blue, black, purple, cyan, and brown), and homologous gene segments from different viruses are shown as different shapes. Nucleotides with low-NP-binding may form secondary structures which are necessary for the incorporation of vRNP. The favored hypothesis of the highly selective genome packaging mechanism relies on the redundant and plastic network of RNA-RNA and potentially RNA-nucleoprotein interactions. Homologous gene segments of IAV compete for incorporation into virions and segment containing matched packaging sequences relative to the background of the virus is packaged preferentially

Influenza virus is a member of the Orthomyxoviridae family and comes in four types, A, B, C and D [1–3]. The virions are enveloped and usually spherical. The influenza virus particle is composed of a viral envelope, matrix proteins and viral ribonucleocapsids (vRNPs). The genome of influenza A virus (IAV) consists of eight single-stranded, negative-sense RNAs that are associated with multiple copies of nucleoprotein and three viral RNA polymerase subunits to form the viral ribonucleoprotein complexes (vRNPs) [4, 5]. Recent studies revealed that the RNP adopts a corkscrew-like morphology with the RNA-dependent RNA polymerase (RdRP) at one end and a loop at the other end [4, 6, 7]. The interaction between NP and the viral RNA was non-uniform and without apparent sequence specificity (Fig. 1) [6–8]. By transmission electron microscopy, the “7 + 1” vRNP organization of a central segment surrounded by seven other vRNPs was observed in influenza virions, including influenza C and D viruses whose genomes are segmented into seven segments [9–11].

**Fig. 2:**
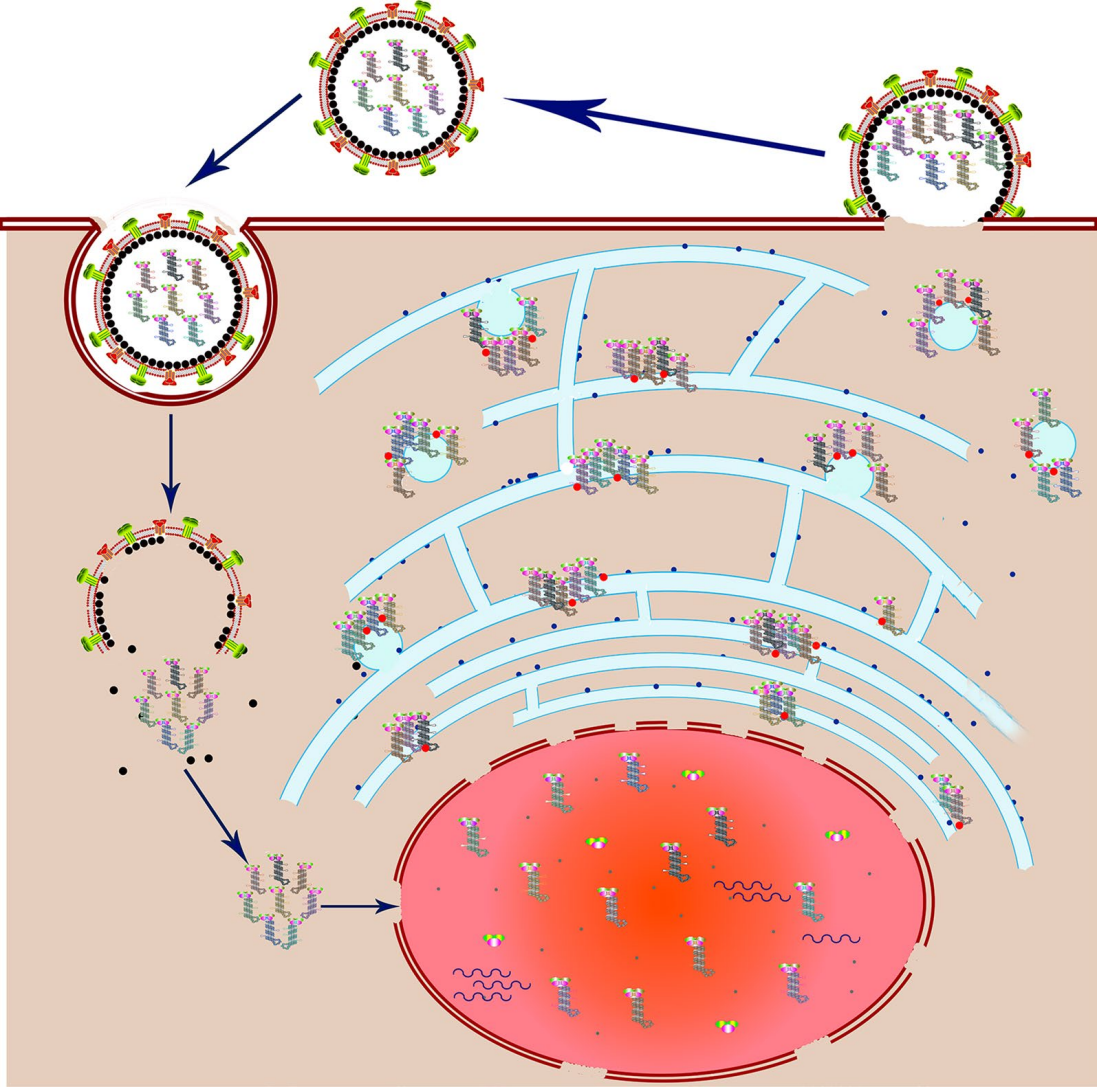
A model for IAV life cycle. IAVs enter host cells by binding the cell surface receptors containing sialic acid. The vRNPs are released into the cytoplasm after the endocytosis and fusion of the viral and endosomal membranes. The incoming vRNAs of IAV remain associated until they are imported into the nuclei of infected cells. The vRNA replicates and transcribes in the nuclei of infected cells and are transported as vRNPs. After exporting from the nucleus, more than one vRNP assembled en route to the plasma membrane and exported from the nucleus as complex. That is, vRNPs together with the Rab11 protein are recruited to the tubulated endoplasmic reticulum (ER) that on irregularly coated vesicles (ICVs). The ICVs loaded with vRNP and Rab11 then bud from the ER and possibly transferred to the plasma membrane. At the final stage, the vRNPs are interconnected at the budding tip of the virion and are oriented perpendicularly. The progeny virions are then released by enzymatic cleavage of the viral receptor mediated by NA protein

After successfully infecting cells, the incoming vRNAs of IAV remain associated until they are imported into the nuclei of infected cells [12]. The vRNA replicates and transcribes in the nuclei of infected cells and are transported as vRNPs (Fig. 2) [13, 14]. De Castro Martin et al. suggested that the transport of vRNP from the nuclei to the plasma membrane primarily depends on the endoplasmic reticulum (ER). When IAV infects the cells, the endoplasmic reticulum (ER) extends throughout the cytoplasm [15]. After exporting from the nucleus, more than one vRNA segments (but not all eight vRNA species) assembled en route to the plasma membrane and exported from the nucleus as complex [12, 16]. Together with the Rab11 protein, the individual and/or sub-bundles vRNP are recruited to the tubulated endoplasmic reticulum (ER) that on irregularly coated vesicles (ICVs). The ICVs loaded with vRNP and Rab11 then bud from the ER and the vRNPs that released from ICVs possibly transferred to the plasma membrane in a touch-and-go process [15]. At the final stage, the vRNPs are interconnected at the budding tip of the virion and are oriented perpendicularly [9, 17], then the progeny virions are released by enzymatic cleavage of the viral receptor mediated by NA protein (Fig. 2) [13, 14].

### Most influenza virions incorporate one copy of each segment

The fragmented nature of the IAV genome confers significant evolutionary advantages to the virus by allowing for easy exchange of gene segments when two or more influenza viruses infect the same cell. However, it undeniably complicates virion assembly, as a nascent influenza virus particle must incorporate at least one copy of each vRNA in order to become replication-competent and thus fully infectious. Inagaki et al. found that the two virus-like RNAs bearing the same terminal sequences competed for incorporation into virions [18]. Based on multicolor single-molecule fluorescent in situ hybridization (FISH), Chou et al. studied the composition of viral RNAs at single-virus particle resolution [19]. The results demonstrated that most of the virus particles package a full set of gene segments and only one copy of each RNA segment is packaged per virion [18, 19].

The genome of influenza C and D virus (ICV and IDV) consists of only seven vRNAs. However, most of the ICV and IDV packaged eight RNPs arranged in the “7 + 1” pattern [10]. The influenza A viruses (IAV) that fail to package the full genome and are noninfectious also exist [20]. Experiments of Nakatsu et al. demonstrated that some influenza A and B virion packaged vRNPs less than eight [21]. Su et al. found that destroying the ubiquitination in M2 of IAV resulted in the production of defective virion particles that lacked vRNPs [22]. These results suggested that the mechanism of influenza virus genome-packaging is flexible and the vRNP number that incorporated by influenza virions is variable [21, 23].

### The random-incorporation and selective-incorporation model

Two different models have been proposed for packaging the RNPs into newly assembling virus particles: the random-incorporation model and the selective-incorporation model [24–26]. The random-incorporation model suggests that each viral RNA segment possesses a common structural feature that allows random incorporation of RNA segments into virions. The selective-incorporation model suggests that unique packaging signals present in vRNAs lead to the incorporation of a set of all eight RNA segments into a virion [13]. Modern day instrumentation as well as biochemical assays and viral assays have provided much evidence to support the selective-incorporation mechanism model of the influenza A genome [9, 26, 27].

For example, Yutaka Fujii et al. generated seven and six segment viruses in the background of A/WSN/33 (H1N1) virus and found that the efficiency of infectious virion production is proportional to the number of different vRNA segments, indicating that vRNA segments contribute individually to virion production [28]. Octaviani et al. coinfected MDCK cells with a swine-origin H1N1 and an avian H5N1 virus. Among 59 viral clones the author examined, there were only 33 different genotypes, much less than 254 in theory. And 15% of the viral clones obtained all of their genes from the H5N1 virus [29]. More recently, Cobbin et al. demonstrated that PB1 gene of A/Udorn/307/1972 (Udorn, H3N2) was preferentially co-packaged with NA gene of the same virus when two NA genes existed [30]. All these data suggested that the genome incorporation of IAV is selective (Fig. 1).

### Packaging signals of different segments

**Fig. 3:**
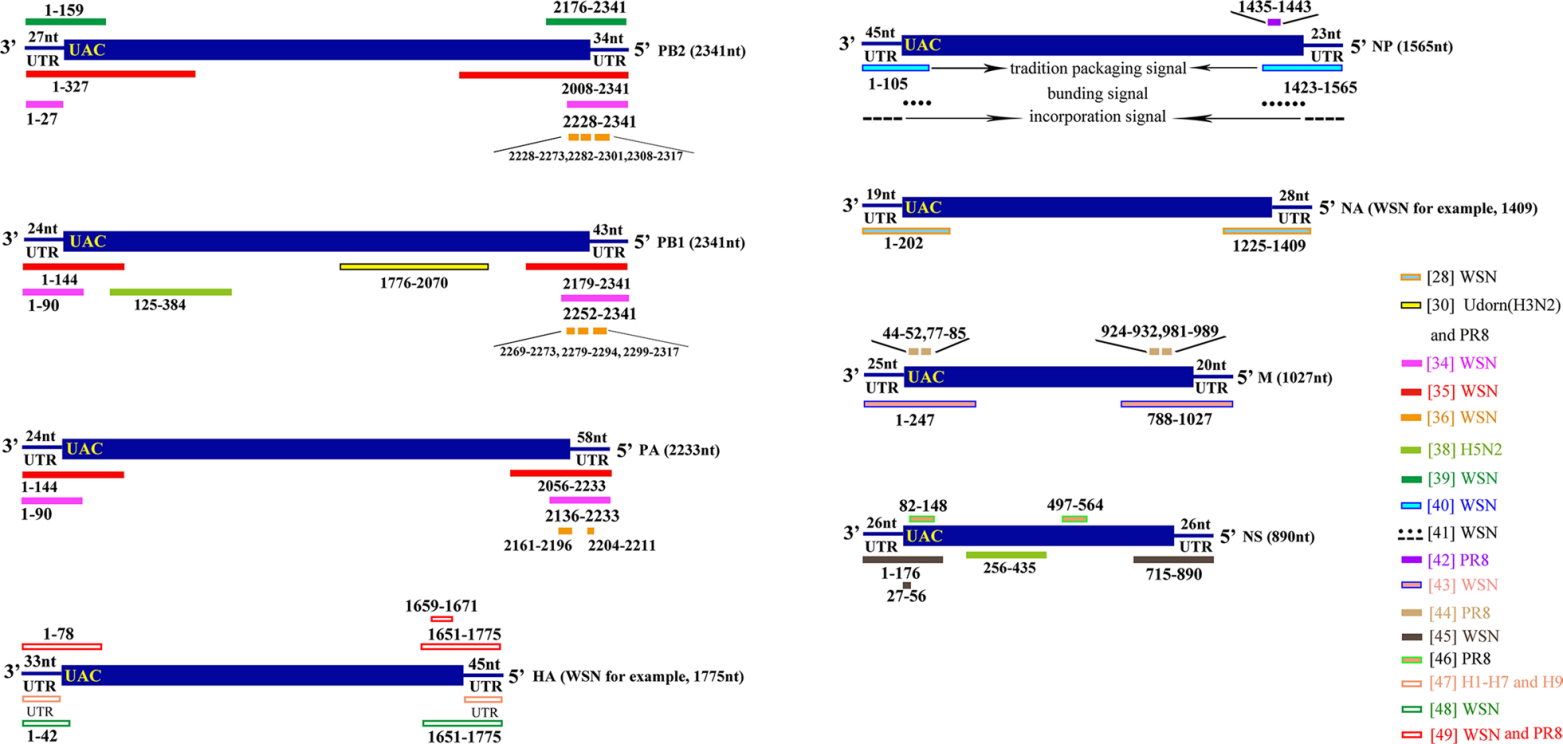
Schematic diagram of the sequences involved in the packaging of influenza A viruses. All segments are shown in the negative-sense orientation and are numbered according to the conventional representation from 3′ to 5′. The sequences necessary for the incorporation of each segment are color-coded and the references plus the corresponding viral strains are listed on the right

The genome vRNAs of IAV share the same organization: a central open reading frame (in the antisense orientation) flanked at both ends by untranslated regions (UTRs) (19–58 nts) [4, 31, 32]. The UTRs of IAV genes consist of two parts: the motif sequences which are highly conserved among viral strains and among the eight segments themselves, located at the 3′ and 5′ termini of every segment; and the segment specific noncoding regions (ssNCRs), the length and sequences of which are specific to each vRNA and between species [26, 33, 34]. The earlier studies identified that the packaging signal consists of a stretch of noncoding regions and the adjacent coding regions at the 3′ and 5′ ends of each vRNA. Whereas, recent studies found that specific sequences within the internal coding regions also play important roles in the genome packaging of influenza A virus (Fig. 3).

#### Packaging signals of the PB2, PB1, and PA genes

Both end coding regions of the WSN PB1 and PA genes are required for genome incorporation and virion formation [35]. Studies from Liang et al. showed that in addition to UTRs, 40-nt at the 5′ end and 66-nt at the 3′ end of the coding sequences of vRNA are minimum requirements for efficient packaging of the PA or PB1 vRNAs of WSN virus [34]. They also found that mutations within the 5′ end coding sequence of WSN PA or PB1 vRNA had more defective impact on packaging efficiency of corresponding vRNA than that within 3′ end coding sequences [36]. More recently, Cobbin et al. found that in competitive plasmid transfection experiment, the PB1 gene of A/Udorn/307/1972 (Udorn, H3N2) cosegregated with the NA gene of the same virus other than the NA gene of A/Puerto Rico/8/1934 (PR8, H1N1) [30]. Analysis revealed that the co-packaging was directed through the internal coding region of the PB1 gene, and the nucleotides 1776–2070 of the PB1 were recently defined as crucial for this preferential selection [30, 37]. With an avian H5N2 virus A/Finch/England/2051/91, Gavazzi et al. identified interaction regions that involved in genome packaging between PB1 and NS genes. They found that the interaction regions of PB1 positioned at 125–384 nt, and the region of NS located at 256–435 nt [38]. These results imply that packaging sequences are viral specific.

Besides the UTR regions, the packaging of the PB2 segment does not require 3′ end coding sequences, but any perturbation of the 5′ end 80-nt coding sequences substantially decreases packaging efficiency [36]. However, Muramoto et al. found that the 3′ end of the PB2 vRNA is crucial for efficient virion incorporation [35]. Afonso et al. revealed that both 3′ and 5′ terminal coding sequence of the WSN PB2 vRNA promoted the incorporation of the minigenomes [39]. This discrepancy may be due to their different assay systems: Yukiko Muramoto et al.replaced the PB2 segment with the GFP gene flanked by the UTR and portions of the coding regions derived from both termini of the PB2 vRNA, whereas Liang et al. generated viruses with GFP reporter genes in the presence of their corresponding authentic vRNA [35, 36].

#### Packaging signal of the NP gene

It has been reported that the packaging signal of NP segment of WSN includes the two terminal UTRs together with 3′ and 5′ terminal coding sequences of NP vRNA that are 60-nt and 120-nt, respectively [40]. Goto et al. demonstrated that in the absence of the other seven vRNAs, the 3′ and 5′ untranslated regions (UTRs) were sufficient for the packaging of NP vRNA. In the presence of the other seven vRNAs, the terminal coding sequences are necessary for both the incorporation efficiency of the recombinant NP vRNA and that of the other vRNAs [41]. The author identified the UTRs as the “incorporation signal” and the terminal coding sequences as the “bundling signal”. By introducing synonymous mutations into highly conserved codons and non-conserved codons in the terminal regions of PR8 NP segment, Hutchinson et al. found that most mutational disruption in NP packaging signals were well tolerated. However, the 5′-terminal codons 464–466 were identified to reduce the packaging level of both NP and PA genes without impacting their vRNA synthesis [42].

#### Packaging signal of the M gene

A 222-nt sequence in the 3′ end coding region and a 220-nt sequence in the 5′ end coding region plus UTR regions at both ends of the M vRNA support the most efficient packaging of this vRNA segment into WSN influenza virions [43]. Ozawa et al. demonstrated that coding region deletions in both ends of the M vRNA significantly decreased the incorporation efficiency of the M gene. Besides, the 27-nt sequence (at positions 979–1005) can be highly diverse without disrupting efficient virus replication [43]. Moreover, synonymous changes within the packaging signals of PR8 M gene showed that the highly conserved codons in this region were closely correlated with the virion assembly and genome packaging. However, similar alterations to nonconserved codons had little effect [44].

#### Packaging signal of the NS gene

Fujii et al. report that at least 150-nt coding regions at both ends of the NS vRNA together with noncoding sequences form a structure that is essential for efficient packaging of WSN vRNA [45]. And the 3′ end sequence had a more drastic effect on vRNA incorporation [45]. Among the regions, the first 30-nt of the 3′ end coding region is critical not only for efficient NS vRNA incorporation but also for virus replication [45]. More recently, Vasin et al. predicted the secondary structures of NS positive-sense RNA. They found that the secondary structure at 82–148 and 497–564 regions were some host and lineage-specific [46]. Further studies with the PR8 NS gene demonstrated that and the structure in 82–148 region would be affected by mutations G123A and A132G, which would influence the protein expression of NS1 [46]. The 82–148 nucleotides fall in the packaging signals mentioned above, whereas, there’s little data demonstrating the relationship between the 497-564nt region and the incorporation of vRNA. However, this region still deserves our attention.

#### Packaging signals of the HA and NA genes

The hemagglutinin (HA) and neuraminidase (NA) segments are both subtype-determinant genes, the segment-specific NCRs of which are subtype specific in sequence and length [47]. Research from Lili Zhao et al. showed that segment specific noncoding regions play an important role in incorporation of HA (H1 to H7 and H9) vRNA. Importantly, noncoding sequences in the 3′ end of HA vRNA are more crucial than those in the 5′ end [47]. The minimal HA (A/WSN/33 (H1N1)) packaging regions identified by Watanable et al. included 9-nt in the 3′ end and 80-nt in the 5′ end coding sequence of the vRNA together with UTRs at both ends [48]. However, Marsh et al. failed to generate a virus with GFP flanked by the packaging region mentioned above. Only with either 45-nt or 60-nt derived from the 3′ end and 80-nt from the 5′ end coding sequence could the virus with the GFP reporter gene be successfully packaged [49]. It’s known that intertypic reassortment has never occurred between influenza A and B viruses. However, Baker et al. successfully obtained the recombinant PR8 virus that carrying HA and (or) NA genes of influenza B virus through appending the respective packaging signals of influenza A virus glycoproteins to influenza B virus HA or NA gene. These results suggested that packaging signals of HA gene are required for effective segment incorporation, and its incompatibilities partly accounted for the inhibition of the intertypic reassortment [50].

Both the 3′ and 5′ end coding regions of NA vRNA are crucial for efficient incorporation of the vRNA [28, 51]. Fujii et al. found that besides the two terminal UTR regions, at least 183-nt in the 3′ end and 157-nt in the 5′ end of the NA coding region are needed for maximal incorporation efficiency of vRNA into virions. Comparatively, the 3′ end coding region plays a more important role [28].

### The conservation of packaging signals

Studies demonstrate that untranslated regions of the IAV genome vRNA are more conserved, having a lower evolutionary rate compared to coding regions [52, 53]. Marsh et al. identified the conserved regions in the polymerase gene segments by analyzing approximately 600 vRNA from avian IAV per segment. They found that the conserved nucleotides are located within the terminal packaging signals and the identified regions also extend to other subtypes of viruses, for example, human IAVs [54]. Gog et al. found that most codons showed very little synonymous variation within terminal packaging sequences among identical segments and among different influenza viruses [55]. But not all the codes in termini are conserved. Hutchinson et al. demonstrated that mutations of the highly conserved codons in packaging signals of M gene reduced virus growth and showed defects in virion assembly and genome packaging. However, mutation in codons that are nonconserved had little effect on the characteristics of the virus [44]. Moreover, there are some conserved codons formed clusters in the middle of coding regions that unidentified functions, most notably in the PA gene [55]. Besides, Gavazzi et al. identified that some interactive regions between PB1 and NS genes is not widely conserved among IAVs [38].

Nevertheless, strain-specific differences exist in packaging signals. As studies have shown that the codons that appear to be important for vRNA packaging in WSN are not in PR8, suggesting that not all packaging signals are conserved [26, 54]. Fujii et al. showed that except the 16–20 nt, the packaging process of NS gene did not absolutely require specific sequences at positions 16–35 nt [56]. It has been reported that nucleotide length of the coding regions within packaging signals was as important as the nucleotide sequences [39, 43]. Zhao et al. observed that mutant viruses possessing similar lengths of the ssNCR to that of the wild-type virus replicated to a level close to that of the wild-type virus, while the other mutant viruses with either shorter or longer ssNCRs showed greater reductions in virus replication efficiency [47].

Together, these results suggest that the incorporation of vRNA is not critically dependent on the wild type nucleotide sequences, and the secondary structure may play a more important role in the process [57]. A conserved RNA pseudoknot structure, predicted in the 5′ end of the NP packaging signal region, was shown to affect virus replication [58]. Stem-loop and hairpin structures that are extremely conserved were predicted within the 3′ and 5′ ends of M packaging signal regions [59, 60]. The viruses with structure-disrupting mutations exhibited lower replication efficiencies and a significantly reduced median plaque size relative to the wild-type virus; when the structure was restored, the mutant virus could replicated at levels comparable to the wild-type virus [59].

### Compatibility of packaging signals between the 3′ and 5′ ends

The motif sequences in the 3′ and 5′ ends of each IAV vRNA are partially complementary to each other. They can form a bulged duplex “corkscrew” structure which is essential for vRNA transcription and replication [61–63]. Therefore, is compatibility required between other parts of the packaging signals at the ends of vRNA? Zhao et al. generated a series of mutant HA plasmids in the context of the WSN virus, that is, one end of the H1-UTR was replaced with the corresponding UTR of other subtype-specific IAV (H2 to H7 and H9), while the other end was unchanged. The results showed that all of these single-end HA UTR substitution viruses were successfully rescued and could replicate efficiently [47]. Liang et al. constructed six hybrid GFP plasmids that carried mismatched 5′ and 3′ packaging signals that were derived from two different vRNAs of the polymerase gene segments (e.g. the 5′ packaging signal from PB2 and the 3′ packaging signal from PB1 or PA). Interestingly, all of these artificial reporter vRNAs were poorly packaged [34]. The different results found by Zhao and Liang may be due to the methods and segments difference.

### Packaging signals determine IAV replication and virion incorporation efficiency

Replacing the wild-type ssNCRs at both ends of the WSN HA vRNA with the corresponding ssNCRs of the H2, H3, H5, and H9 subtypes resulted in lower replication efficiency of the mutant viruses than the wild-type virus, especially at earlier time points post infection. And the 3′ end ssNCR substitution viruses demonstrated a more drastic reduction [47]. Barman et al. successfully improved the replication of A/Anhui/1/2013(Anhui/1, H7N9) influenza vaccine virus in eggs by more than a twofold higher titer than that of wild-type by constructing a chimeric gene with the coding sequence of the A/Anhui/1/2013 (Anhui/1, H7N9) NA vRNA and the packaging signals from the PR8 NA vRNA [64]. Recently, stem-loop structures were found at the terminal packaging sequences in both M and PB2 vRNA. Disrupting the predicted secondary structures significantly attenuated the infectivity of the mutant virus and increased the production of defective virus particles [60, 65]. Spronken et al. obtained similar results by studying the conserved hairpin structure in the 967–994-nt of the M vRNA using a compensatory mutagenesis approach [59]. These data suggest that packaging signals of IAVs are very important for efficient viral replication and incorporation.

### Roles of packaging signals in genetic reassortment

Extensive evidence indicates that protein incompatibility among both polymerase subunits and packaging signals of vRNAs are restricting factors for reassortment between two viruses [50, 66–68]. Several reports indicated that in natural or experimental competitive situations, the number of reassortant viruses was very low, and the reassortant genotypes were not random [26, 67, 69]. Essere et al. reported that an avian HA segment of H5N2 could not be incorporated alone into the H3N2 genetic background when competing with the HA segment of the H3N2 virus. However, introducing 5′ and 3′ packaging sequences of the H3N2 HA into an otherwise H5N2 HA backbone or introducing five silent mutations into the H3N2 M segment was sufficient to disrupt this limitation [69].

Recent studies found that the HA segment containing matched packaging signals relative to the background of the virus was packaged preferentially, but there was no preference for homologous packaging signals for the NA and NS segments [70]. These results indicate that the NA and NS segments could move between human H3N2 and H1N1 lineages without restrictions of packaging signal mismatches, while the movement of the HA segment would be limited [70]. By swapping the packaging sequences of NS and HA gene and destroying the intrinsic packaging sequences of the two segments, Gao et al. created a chimeric HA and NS segment with packaging signals of the NS and HA gene. The rewired viruses could be rescued successfully and replicate efficiently, but they lost their ability to independently reassort their HA or NS gene [71].

All of these researches suggested that IAV packaging signals play a critical role in mediating the reassortment between different viruses, and the importance of packaging signals in the process may be segment dependent. Beyond IAV packaging signals, some amino acids may also be required for packaging of specific RNA segments. Moreira et al. demonstrated that the head and body domain of NP impair genome packaging of influenza viruses; thus, the author referred to this set of amino acids as the “NP packaging code” [72]. Bolte et al. showed that packaging sequence mutation of one vRNA won’t impact the genome packaging of an H7N7 virus, whereas combining the mutation with specific nucleoprotein amino acid will impair the process greatly [73].

### Packaging signals are relative to Hierarchy and cooperation among vRNA segments

A series of studies demonstrated that not all vRNAs are equally important in their role in vRNA incorporation into influenza A virions [35, 49, 54]. A recent study showed that PB2, PA, NP and M play a more crucial role than other vRNAs in the packaging procedure of the PR8 virus [26, 35, 54, 74]. Moreover, reductions in the packaging of the PB2 vRNA reduced incorporation efficiency of PB1, PA, NP, M and NS vRNA in WSN virus significantly, but had little impact on the packaging level of HA or NA vRNA [54]. Nevertheless, another experiment showed that when lacking the HA segment, the growth of the virus was reduced and the packaging of other segments was impaired in WSN virus, particularly the PA, NP, NA, M and NS vRNAs [49].

For all IAV segments, deletions or mutations in their packaging regions affect vRNA incorporation efficiency, not only for the segment in which they reside, but also for other segments. For the three polymerase segments, this impact was most prominent for the PB2 gene [35]. A key region of the open reading frame (1659–1671-nt) in both the WSN and PR8 virus HA segments was identified [49]. Mutations in the region resulted in significant reductions in the packaging of the HA and other vRNAs. The most affected segments were PB1 vRNA in the WSN virus and NA and M vRNAs in the PR8 virus [49]. These results indicate that some cooperation exists between vRNAs and virus-specificity exists [54].

Actually, the favored hypothesis of the highly selective genome packaging mechanism relies on RNA-RNA or protein-RNA interactions (Fig. 1) [17, 34, 35]. As early as 2011, Fournier et al. found that the eight vRNPs of a human H3N2 IAV are interconnected in a “transition zone” at the budding tip of virions [17]. Further studies showed that the vRNAs formed a single network of intermolecular interactions in vitro and the known packaging signals are identified as the strongest interactions [17, 75]. Similarly, for the H1N1 WSN virus, most of the inter-segment interactions were mediated by their 3′ and 5′ terminal packaging signals [76]. Another study demonstrated that the interaction network also existed in an avian H5N2 IAV. However, both the interaction network and the involved sequences of each vRNA varied from those of the human H3N2 IAV mentioned above [77]. By studying interactions between PB1 and NS segments of the avian H5N2 IAV, they further proved that the interaction identified in vitro takes place in infected cells and was crucial for viral replication, and genome packaging and reassortment. Noticeable was that the sequences identified as interaction not located in the packaging signals [38].

### Different opinions about IAV genome packaging

It’s not difficult to find that the identified sequences required for the genome packaging are varied from different research groups, and some relevant conclusions seem conflict. Moreover, other recently emerging ideas that are contrary to conventional wisdom are also notable. For example, experiment from Dadonaite et al. revealed that the RNA-RNA interactions are extensive, redundant and complex, rather than a finite set of discrete interactions between packaging signals [57]. Bolte et al. suggested that the packaging of IAV vRNA is governed by the redundant and plastic network of RNA-RNA and potentially RNA-nucleoprotein interactions [73]. Contrary to the classical model that nucleoprotein binds vRNA as a uniform pattern of “beads on a string” [78–80], the nucleoprotein binds vRNA non-uniformly and without apparent sequence specificity. For example, some sites are enriched and some sites are poor in nucleoprotein association [6, 7, 80]. The sequences with low-NP-binding, including both internal sequences and the traditional packaging signals of the segment, are necessary for virus propagation [6, 7]. Different from the RNA-RNA molecular recognition mechanism in genome packaging of IAV, Venev et al. proposed another hypothesis. They generated model 8-segment virions by using monte carlo simulations. The results demonstrated that selective packaging of the IAV genome can be achieved by self-repulsion of identical segments rather than by molecular recognition [81].

## Conclusion

The mechanisms that govern IAV genome packaging have been extensively studied. However, the precise mechanisms of the IAV genome incorporation remain unclear. Some results from different research teams are varied and even “conflict”. For instance, the PB1 3′ packaging signals of PR8 play roles for the incorporation of PR8 PB1, whereas the central coding sequence is responsible for the coselection of a H3N2 IAV’s PB1 and NA gene [30, 37]. Some studies demonstrated that packaging signals play important roles in IAV genome incorporation, reassortment and viral replication [47, 64, 69–71, 82]. But some studies argue that interactions between RNA-RNA and RNA-nucleoprotein are key determinants of IAV genome packaging and the packaging signals are redundant [57, 73].

It’s notable that the many different results were based on different IAV genotypes or virus strains. And the genome packaging of IAV involves a series of complex steps. Thus, the studies are of reference value to study the precise mechanisms of the IAV genome incorporation. At present, the highly selective nature of the genome packaging is widely admitted though the exact mechanisms of the IAV genome incorporation need more extensive researches. However, the puzzle will be revealed as research progresses.

## Data Availability

Not applicable.

## Abbreviations

IAV: Influenza A virus
vRNPs: Viral ribonucleocapsids
RdRP: RNA-dependent RNA polymerase
UTR: Untranslated regions
ssNCRs: Segment specific noncoding regions
ER: Endoplasmic reticulum
ICV: Irregularly coated vesicles
vRNA: Viral RNA
